# Association between growth differentiation factor 15 levels and gestational diabetes mellitus: A combined analysis

**DOI:** 10.3389/fendo.2023.1084896

**Published:** 2023-01-20

**Authors:** Yi-Cheng Lu, Song-Liang Liu, Yu-Shan Zhang, Fei Liang, Xiao-Yan Zhu, Yue Xiao, Jing Wang, Cong Ding, Sudipta Banerjee, Jie-Yun Yin, Qiu-Ping Ma

**Affiliations:** ^1^ Taicang Affiliated Hospital of Soochow University, The First People’s Hospital of TaiCang, Soochow University, Suzhou, China; ^2^ Jiangsu Key Laboratory of Preventive and Translational Medicine for Geriatric Diseases, School of Public Health, Medical College of Soochow University, Suzhou, China; ^3^ Suzhou Center for Disease Prevention and Control, Suzhou, China; ^4^ Department of Sample Application and Management, Institute of Suzhou Biobank, Suzhou, China; ^5^ Department of Endocrinology and Metabolism, Institute of Post-Graduate Medical Education and Research and Seth Sukhlal Karnani Memorial Hospital (IPGME & R and SSKM Hospital), Kolkata, India

**Keywords:** gestational diabetes mellitus, growth differentiation factor 15, macrophage inhibiting cytokine-1, meta-analysis, diabetes

## Abstract

**Objective:**

Gestational diabetes mellitus (GDM) is a common glucose metabolism disease occurs in pregnancy that affects both maternal and neonatal health. Recently, increasing studies have attached importance to the relationship between growth differentiation factor 15 (GDF-15) and GDM, but the results were inconclusive. Therefore, we conducted a meta-analysis to examine the association between GDF-15 and GDM.

**Materials and methods:**

A systematical search was performed in Gene Expression Omnibus (GEO), PubMed and Google Scholar till Oct 27, 2022. We first calculated the mean and standard deviation of GDF-15 expression levels from the included eligible datasets and articles. Then, a meta-analysis was conducted to depict the difference in *GDF-15* mRNA or GDF-15 protein expression between case and control groups by using conservative random effect model. Moreover, the potential publication bias was checked with the aid of Begg’s test and Egger’s test. Finally, sensitivity analyses were performed by changing the inclusion criteria.

**Results:**

In summary, 12 GEO datasets and 5 articles were enrolled in our study, including 789 GDM patients and 1202 non-GDM pregnant women. It was found that the expression levels of *GDF-15* mRNA and GDF-15 protein in late pregnancy were significantly higher in GDM patients compared with non-GDM pregnant women, with the standard mean difference (SMD) and 95% confidence interval (95% CI) of 0.48 (0.14, 0.83) and 0.82 (0.32-1.33), respectively. Meanwhile, a slightly weakened association between GDF-15 protein levels and GDM was also observed in the middle pregnancy, with SMD (95% CI) of 0.53 (0.04-1.02).

**Conclusion:**

In all, our results suggested that the expression levels of GDF-15 were significantly higher in GDM patients compared with non-GDM pregnant women, especially in the late pregnancy.

## Introduction

Gestational diabetes mellitus (GDM) is defined as diabetes or impaired glucose intolerance occurring for the first-time during pregnancy. It was estimated that the incidence of GDM ranged from 6.1% to 15.2% in the whole world (1). In China, the incidence of GDM was up to 14.8% (2). Without proper diagnosis and treatment, GDM can increase the risk of maternal complications (including gestational hypertension, urinary tract infection, and polyhydramnios) (3), as well as infant morbidities (such as macrosomia, erythrocytosis and hypoglycemia) (4). What’s more, GDM mothers and their offspring are more likely to develop obesity, type 2 diabetes mellitus (T2DM), and cardiovascular diseases (CVD) in later life (5–7). Therefore, it is of great clinical and health significance to explore the pathogenesis of GDM, and therefore to help in early screening, early diagnosis and thus early intervention of GDM to ensure maternal and fetal health.

Growth differentiation factor 15 (GDF-15) is a divergent member of the transforming growth factor-β superfamily (8). GDF-15 was reported to be an inflammation-induced central mediator of tissue tolerance (9). Levels of GDF-15 are markedly elevated in inflammatory disease states (10). GDF-15 was also found to suppress the intake of high-fat diets in animal models (11–13). Pharmacological treatment of recombinant GDF-15 proteins could reduce body weight and improve glucose tolerance in obese rodents and primates (12, 14). Moreover, metformin, the most commonly prescribed medication for T2DM, was revealed to achieve weight loss and glycemic control by stimulating the secretion of GDF-15 (14). Meanwhile, epidemic studies suggested that GDF-15 were associated with glucometabolic diseases (15), including T2DM (16).

GDM shares many features with T2DM in pathogenesis, for instance, glucose metabolism and insulin resistance (17, 18). Moreover, obesity, which could cause low-grade activation of inflammation and dysregulation of adipokines, is a major risk factor for these two diseases (19, 20). Along with observed associations of GDF-15 with T2DM, there was also an assumption that GDF-15 had a role in the development of GDM. However, the sample size of studies shed light in this field was relatively small and the results were far from conclusive. For example, two Norway studies reported that the GDF-15 levels were comparable between GDM patients and non-GDM pregnant women (21, 22), while Banerjee et al. found that GDF-15 concentrations at 24-32 weeks of gestation were significantly higher in GDM versus age-matched pregnant controls (23). Besides, Li et al. revealed a positive correlation between levels of GDF-15 in middle pregnancy and GDM (24), while Tang et al. reported that serum GDF-15 levels in the late pregnancy instead of the middle pregnancy were positively correlated with glucose metabolism (25).

Based on these inconsistent results in the current literature, we aimed to conduct a meta-analysis through search of GEO datasets and relevant literatures to figure out the association between GDF-15 and GDM.

## Materials and methods

### Data acquisition and search strategy

**Figure 1:**
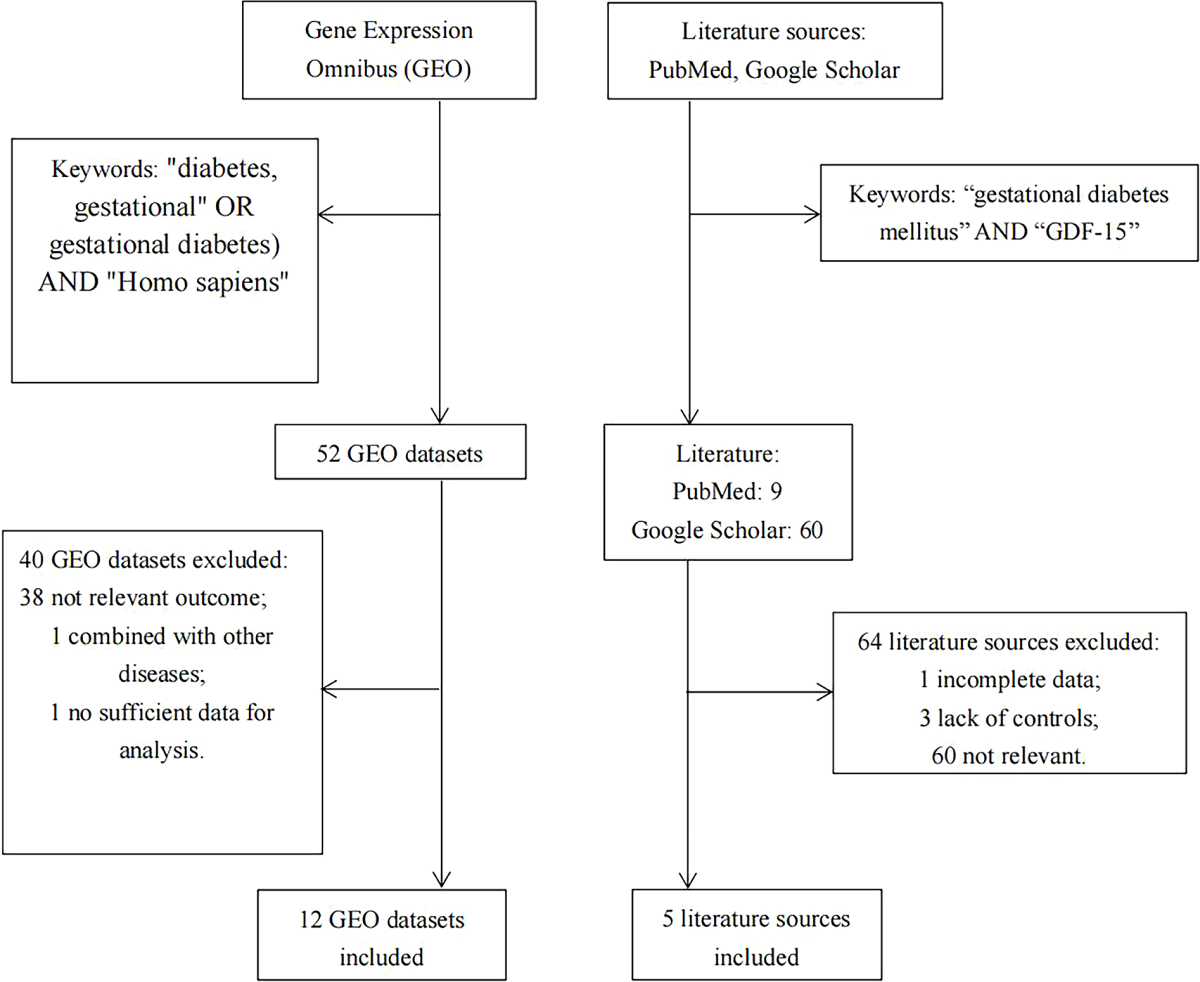
Flow diagram for literature selection.

Relevant databases were searched in the Gene Expression Omnibus (GEO; https://www.ncbi.nlm.nih.gov/geo/) for the meta-analysis up to Oct 27, 2022, using the following subject terms (“diabetes, gestational” OR “gestational diabetes”) AND “Homo sapiens”. Then, suitable literatures were manually searched in PubMed and Google Scholar, using the keywords of “gestational diabetes mellitus” AND “GDF-15”. The detailed search strategy was illustrated in **Figure 1**.

### Inclusion and exclusion criteria

Studies were considered eligible if they met the following criteria (1): the expression levels of GDF-15 were compared between GDM patients and non-GDM pregnant women; (2) the mean and standard deviation (SD) of GDF-15 should be extracted or calculated; (3) only human samples could be included; (4) because little evidence was reported for early pregnancy, thus only data from the middle and late pregnancy were included if GDF-15 was evaluated at different gestational weeks in one study. Data collected after delivery was categorized into the late pregnancy group. Exclusion criteria were as follows: (1) patients enrolled in the study have developed diabetes before pregnancy; (2) studies were performed in animals or cell lines; (3) samples have overlapped with other studies. One article was excluded because of insufficient data for analysis (26).

### Quality control and data extraction

Two authors (Yicheng Lu and Yushan Zhang) independently assessed the eligible datasets according to the inclusive and exclusive criteria, and any divergence related to study inclusion was settled within the team. The following information of each study was extracted: the first author, country of origin, publication year, study subjects (disease status and sample type), the exclusion criteria of case/controls, as well as expression values, means, and SD of GDF-15.

In GSE65737, 30 pairs of GDM macrosomia and normal controls were divided into three subgroups randomly, and the umbilical cord vein blood from each subgroup was mixed and hybridized to a microarray. Therefore, the mean and SD were calculated based on the data from the pooled subgroups. In GSE203346, the dataset simultaneously collected samples of both placenta from pregnant women and umbilical cord blood, our study only included data from maternal placenta.

### Statistical analysis

We first calculated the mean and SD of GDF-15 expression levels from the included eligible datasets and articles. If the included literature reported median and interquartile range, we used the compiled formula online calculator provided by Wan et al. (27) and Luo et al. (28) (https://www.math.hkbu.edu.hk/~tongt/papers/median2mean.html) to transform these values into mean and SD. Then, a meta-analysis was conducted to depict the difference in GDF-15 expression between case and control groups. Forest plots were used to express the pooled standard mean difference (SMD) and 95% confidence interval (95% CI). Considering the included studies used various tissues and platforms for mRNA quantification, we used conservative random effect model for data combination. Moreover, the potential publication bias was checked with the aid of Begg’s test and Egger’s test. Finally, sensitivity analyses were performed by excluding one study in turn to assess possible biases caused by a single study, or by adopting different inclusion criteria. All analyses were conducted with the assistance of SAS (SAS Institute Inc., NC, USA) and STATA (STATA Corporation, College Station, TX, USA).

## Results

### Basic characteristics of included studies

As illustrated in **Figure 1**, there were 5 articles (22–25, 29) and 12 GEO datasets finally met the inclusion criteria. For the 5 articles measured serum GDF-15 protein levels, 640 GDM patients and 880 controls were recruited during their middle or late pregnancy in China, India, and Turkey. Among the 12 GEO datasets including 149 GDM patients and 322 controls detecting *GDF-15* mRNA, the vast majority were conducted at delivery; in contrast, there was only one relevant dataset (GSE154377) with 7 GDM patients and 9 controls who were recruited in their middle pregnancy. Meanwhile, the diagnostic criteria for GDM varied in studies, 3 datasets (including GSE65737, GSE103552 and GSE203346) and 1 article (23) used criteria from the International Association of Diabetes and Pregnancy Study Groups, 1 dataset (GSE154377) and 1 article (29) followed the American College of Obstetricians and Gynecologists, 3 articles (22, 24, 25) referred to the World Health Organization, while the remained studies (including GSE49524, GSE70493, GSE87295, GSE51546, GSE128381, GSE150621, GSE154414 and GSE194119) did not mention the diagnostic criteria. What’s more, 5 GEO datasets (including GSE70493, GSE65737, GSE128381, GSE154377 and GSE203346) and 4 articles (22, 24, 25, 29) excluded preeclampsia or other diseases that may influence GDF-15 expression during sample selection. The detailed information on exclusion criteria of each study was shown in **Supplementary Table 1**.

### Meta-analysis of GDF-15 mRNA expression in GDM patients and controls

**Figure 2:**
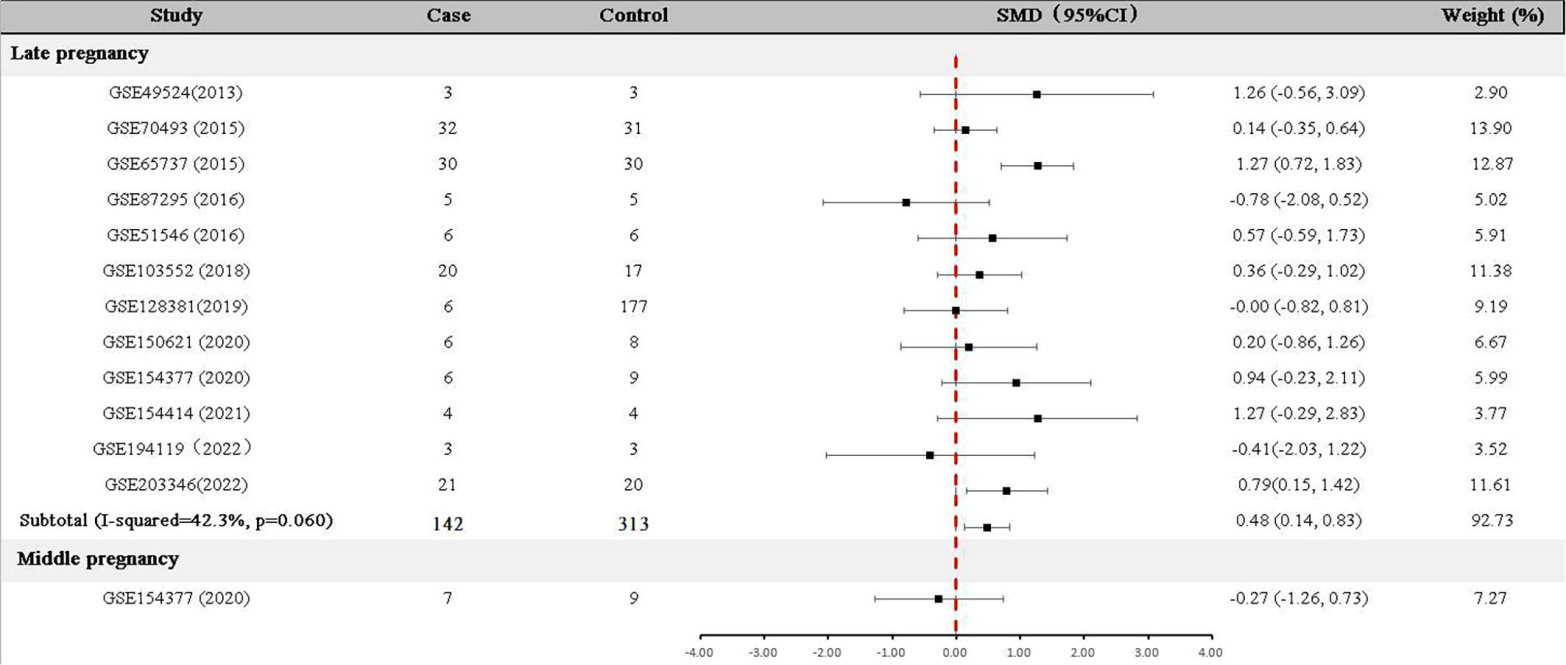
Difference of *GDF-15* mRNA expression between GDM patients and non-GDM pregnant women.

For data collected in the late pregnancy, 12 relevant datasets (GSE49524, GSE70493, GSE65737, GSE87295, GSE51546, GSE103552, GSE128381, GSE150621 GSE154377, GSE154414, GSE194119 and GSE203346) with 142 GDM patients and 313 controls were available (**Table 1**). As shown in **Figure 2**, the expression of *GDF-15* mRNA in the late pregnancy was significantly higher in GDM patients compared with that in controls (SMD=0.48, 95% CI=0.14-0.83). There was only one relevant dataset (GSE154377) that was conducted in the middle pregnancy, reporting that *GDF-15* mRNA was not statistically different between controls and GDM (SMD=-0.27, 95% CI=-1.26-0.73).

**Table 1:** Characteristics of *GDF-15* mRNA expression profiling datasets included in the current meta-analysis between GDM and controls.

Dataset	Measuring time	Country and publication year	Sample type	GDM diagnostic criteria	Platform	Case		Control		Excluding &
						Sample size	Mean ± SD of *GDF-15*	Sample size	Mean ± SD of *GDF-15*	
GSE49524	Delivery	Italy, 2013	Umbilical cord	NA	GPL7020	3	11.410 ± 0.470	3	10.965 ± 0.165	Not mentioned
GSE70493	Delivery	America, 2015	Placental tissue	NA	GPL17586	32	10.774 ± 0.356	31	10.708 ± 0.541	Yes
GSE65737	Delivery	China, 2015	Umbilical cord vein blood	IADPSG criteria, 2018	GPL16956	30	5.485 ± 0.073	30	5.265 ± 0.232	Yes
GSE87295	Delivery	Singapore, 2016	Human umbilical vein endothelial cells	NA	GPL10558	5	624.648 ± 281.441	5	2699.146 ± 3743.309	NA
GSE51546	Delivery	Finland, 2016	Umbilical cord	NA	GPL10558	6	82.418 ± 16.052	6	75.397 ± 6.509	NA
GSE103552	Delivery	Austria, 2018	Primary feto-placental endothelial cells	IADPSG criteria,2018	GPL6244	20	11.975 ± 0.262	17	11.879 ± 0.267	NA
GSE128381	Delivery	Belgium, 2019	Placental tissue	NA	GPL17077	6	18.405 ± 0.230	177	18.405 ± 0.260	Yes
GSE150621	Delivery	America, 2020	Amniocytes	NA	GPL16791	6	4191.167 ± 2453.229	8	3681.250 ± 2649.155	NA
GSE154377	Late pregnancy	America, 2020	Blood	ACOG guidelines, 2018	GPL20301	6	4.167 ± 3.976	9	1.778 ± 1.787	Yes
	Middle pregnancy	America, 2020	Blood	ACOG guidelines, 2018	GPL20301	7	0.714 ± 0.951	9	1.333 ± 2.958	Yes
GSE154414	Delivery	China, 2021	Placental tissue	NA	GPL20301	4	844.421 ± 221.869	4	643.824 ± 31.750	NA
GSE194119	Delivery	China,2022	Cord blood	NA	GPL22120	3	1.351 ± 0.830	3	1.657 ± 0.663	NA
GSE203346	Delivery	Norway,2022	Placental tissue	IADPSG criteria, 2018	GPL24676	21	4.885 ± 1.198	20	3.650 ± 1.885	Yes

IADPSG, the International Association of Diabetes and Pregnancy Study Groups.

ACOG, the American College of Obstetricians and Gynecologists.

NA, not available.

&, preeclampsia or other diseases that may influence GDF-15 expression.

### Meta-analysis of GDF-15 protein expression in GDM patients and controls

**Figure 3:**
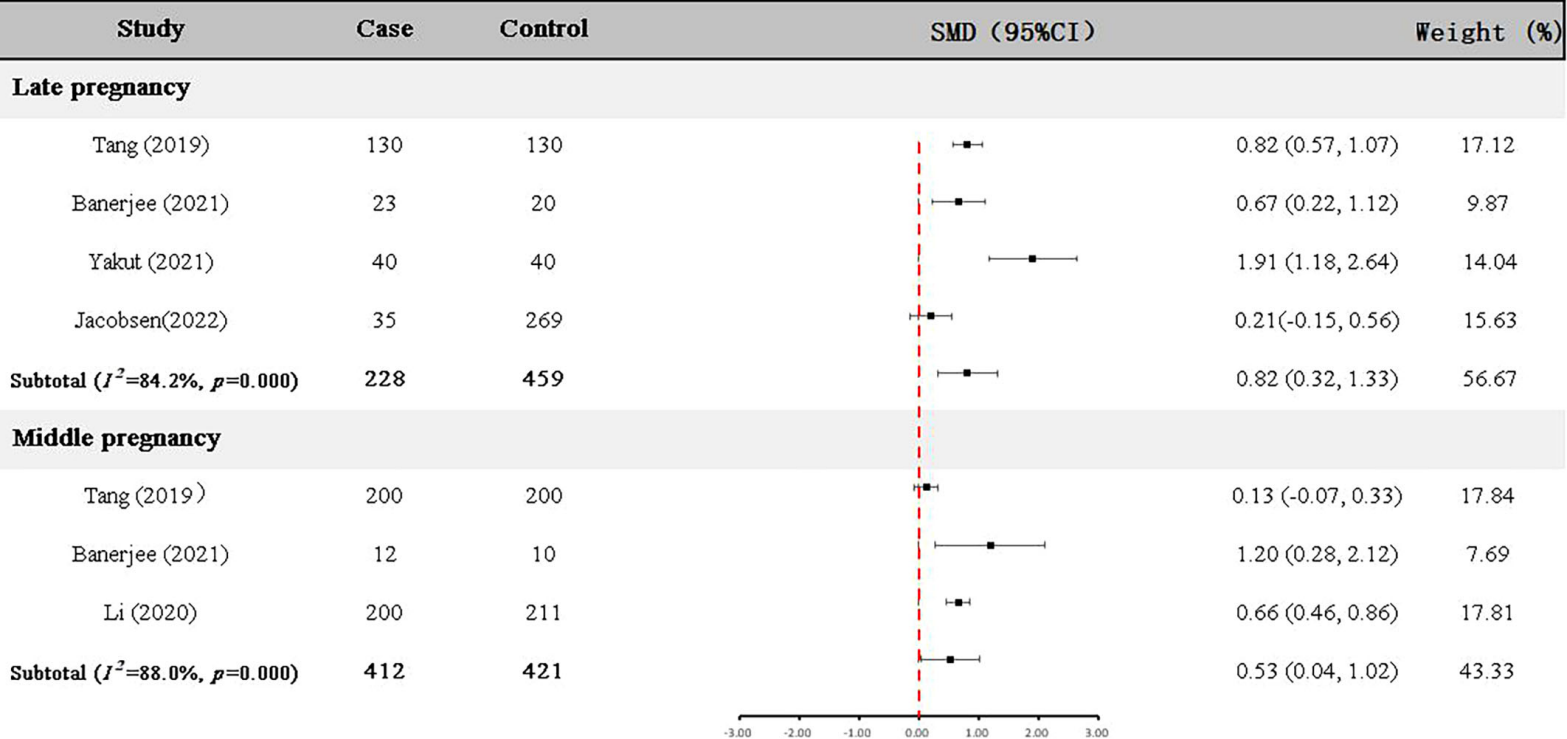
Difference of GDF-15 protein expression between GDM patients and non-GDM pregnant women.

Four articles (22, 23, 25, 29) with 228 GDM patients and 459 controls examined GDF-15 protein in the late pregnancy (**Table 2**). As shown in **Figure 3**, the expression level of GDF-15 protein in GDM patients was statistically higher than that in controls (SMD=0.82, 95% CI=0.32-1.33).

**Table 2:** Characteristics of GDF-15 protein expression profiling datasets included in the current meta-analysis between GDM and controls.

Dataset	Measuring time	Country and publication year	Sample type	GDM diagnostic criteria	Platform	Case		Control		Excluding &
						Sample size	Mean ± SD of GDF-15	Sample size	Mean ± SD of GDF-15	
Tang* (25)	Late pregnancy	China, 2019	Serum	WHO guidelines,2013	Protein	130	114.787 ± 47.627	130	81.381 ± 32.558	Yes
Banerjee (23)	Late pregnancy	India, 2021	Serum	IADPSG criteria, 2018	Protein	23	1091.6 ± 115.4	20	828.5 ± 160.0	NA
Yakut* (29)	Late pregnancy	Turkey, 2021	Serum	ACOG guidelines, 2018	Protein	40	1786.347 ± 3176.192	40	283.604 ± 158.833	Yes
Jacobsen* (22)	Late pregnancy	Norway, 2022	Serum	WHO guidelines, 2013	Protein	35	99036.566 ± 40566.734	269	91134.718 ± 37806.970	Yes
Tang* (25)	Middle pregnancy	China, 2019	Serum	WHO guidelines,2013	Protein	200	32.652 ± 14.748	200	30.868 ± 12.732	Yes
Banerjee (23)	24-28 weeks (middle pregnancy)	India, 2021	Serum	IADPSG criteria, 2018	Protein	12	1002.8 ± 176.3	10	820.5 ± 115.6	NA
Li (24)	24-28 weeks (middle pregnancy)	China, 2020	Serum	WHO guidelines, 2013	Protein	200	18.462 ± 8.023	211	13.941 ± 5.567	Yes

*Original data was median and interquartile range.

WHO, the World Health Organization.

IADPSG, the International Association of Diabetes and Pregnancy Study Groups.

ACOG, the American College of Obstetricians and Gynecologists.

&, preeclampsia or other diseases that may influence GDF-15 expression.

In addition, GDF-15 protein of 412 GDM patients and 421 controls were assessed in the middle pregnancy in three articles (23–25) (**Table 2**). As shown in **Figure 3**, the expression level of GDF-15 protein was slightly elevated in GDM patients compared with controls (SMD=0.53, 95% CI=0.04-1.02).

Meanwhile, we tried to calculated the reference range by using random effect model (inverse variance method) (30). Representing as the 95%CI of the averaged level among controls, the reference range of circulating GDF-15 in late pregnancy was 1913.73-3396.96 pg/ml, corresponding reference range in middle pregnancy was 75.64-126.54 pg/ml.

### Publication bias and sensitivity analyses

Overall, Begg’s test and Egger’s test consistently indicated that there was no publication bias for the mentioned combinations above (all *P* values were above 0.05, shown in **Supplementary Figures S1A, S1B**).

A sensitivity analysis was performed by excluding one study in turn to assess possible biases caused by a single study, the results were basically stable (**Supplementary Figures S2A, S2B**).

We then explored whether the results were changed if we restricted to studies that excluded preeclampsia or other diseases that may influence GDF-15 expression during their sample selection. In the 5 GEO datasets (including GSE70493, GSE65737, GSE128381, GSE154377 and GSE203346), it was found that the expression level of *GDF-15* mRNA in late pregnancy was significantly higher in 95 GDM patients compared with 267 non-GDM pregnant women, with SMD (95% CI) of 0.61(0.09,1.13) (**Supplementary Figure S3**). In three articles enrolled 205 GDM patients and 439 controls in late pregnancy (22, 25, 29), the expression level of GDF-15 protein was significantly elevated in GDM patients compared with controls (SMD=0.57, 95%CI=0.18-0.96) (**Supplementary Figure S4**). However, in the two articles containing 400 GDM patients and 411 controls (24, 25), it was found that the difference of the expression levels of GDF-15 protein in the middle pregancy was not significant between GDM patients and non-GDM pregnant women, with SMD and 95%CI of 0.39 (-0.12, 0.91) (**Supplementary Figure S4**).

## Discussion

By combining expression data of mRNA and protein, we found that GDF-15 was upregulated in GDM patients in different tissues compared with non-GDM pregnant women, indicating that GDF-15 may be used as a biomarker of GDM. It should be noted that one study, which was excluded due to incomplete data for meta-analysis, reported consistent results with ours (26). Similarly, GDF-15 was found to be associated with another common gestation complication [preeclampsia (31)].

We hypothesized that GDF-15 may be compensatively upregulated in GDM, similar to the results reported for other disorders of glucose metabolism (16, 32–34). On one hand, as we mentioned before, GDF-15 appears to maintain systemic energy homeostasis. In pregnant women, GDF-15 was positively related with nausea and vomiting (35), a common gestational condition that may cause low gestational weight gain. Consistently, studies observed that GDF-15 was inversely correlated with maternal BMI and gestational weight gain during pregnancy (36, 37), which are important risk factors of GDM (38). On the other hand, there was evidence that GDF-15 exerted anti-inflammatory role through inhibiting the activation of macrophages (39), while the role of immune activation and inflammation in the pathogenesis of GDM has widely accepted (40). Therefore, GDF-15 may be induced in response to the altered energy metabolism and increased inflammation of GDM.

GDF-15 exerted protective effect in the disorders of glucose metabolism in preclinical studies but possessed paradoxically positive association with such disease in epidemiological surveys. Similar paradoxical results of GDF-15 were also observed in other disease settings. For example, epidemiological studies have demonstrated that higher GDF-15 levels are unfavorably associated with CVD progression and prognosis (41). However, animal studies suggested that GDF-15 was probably a cardioprotective factor (42). For another example, GDF-15 levels are found to be correlated with an increased risk of chronic kidney disease progression (43) or albuminuria in patients with T2DM (44), while animal study suggested that GDF-15 could be reno-protective (45). It also should be mentioned that mendelian randomization studies revealed that there was a null association between GDF-15 and the risk of CVD (46) and T2DM (47), suggesting that GDF-15 was only a biomarker for related diseases instead of a causal factor. Therefore, our results support that GDF-15 could serve as a biomarker of GDM. Nevertheless, whether GDF-15 plays a causal role in the pathogenesis of GDM or is just a bystander, requires further investigation.

Several limitations in our study should be noted. Firstly, it is reported that the level of serum GDF-15 gradually increased during the progression of gestation (48), and the association between GDF-15 and GDM may differ depending on the trimester of pregnancy (25). However, only three articles (23–25) and one study (GSE154377) with a total of 419 GDM patients and 430 controls assessed GDF-15 in the middle pregnancy. Therefore, further studies aimed for the early and middle pregnancy should be encouraged. Secondly, the combined analyses of the current study were based on mean and SD. But three original literatures (22, 25, 29) only reported median, interquartile range or range, the conversion of which to mean and SD may cause some bias. Thirdly, the included studies used different diagnostic criteria for GDM and collected different maternal or fetal tissues. Therefore, we used a conservative random effect model for the combination. Fourthly, tissues collected at delivery was categorized into the late pregnancy group, which may cause some bias. Lastly, as a feature of meta-analysis, our study could not explore the mechanism of GDF-15 in GDM. Further studies are warranted to figure this out.

## Conclusions

Our results suggested that the expression levels of GDF-15 were significantly higher in GDM patients than in non-GDM pregnant women, especially in the late pregnancy, indicating that GDF-15 may act as a biomarker for GDM.

## Author contributions

Y-CL and S-LL searched data and draft the manuscript; JY-Y and QP-M designed the study and revised the manuscript; all other authors contributed in the data extraction or manuscript revision.

## Supplementary material

Supplementary Figure 1 Begg’s funnel plot for the assessment of potential publication bias in the samples (**A**: mRNA; **B**: protein).

Supplementary Figure 2 Sensitivity test of GDF-15 expression between GDM patients and non-GDM pregnant women (**A**: mRNA; **B**: protein).

Supplementary Figure 3 Difference of *GDF-15* mRNA expression between GDM patients and non-GDM pregnant women (excluding preeclampsia or other diseases that may influence GDF-15 expression).

Supplementary Figure 4 Difference of GDF-15 protein expression between GDM patients and non-GDM pregnant women (excluding preeclampsia or other diseases that may influence GDF-15 expression).
