# Supplementary figures and images for: Association between growth differentiation factor 15 levels and gestational diabetes mellitus: A combined analysis
Source: Front Endocrinol (Lausanne). 2023 Jan 20;14:1084896. doi: 10.3389/fendo.2023.1084896 (PMC9895392; doi:10.3389/fendo.2023.1084896)

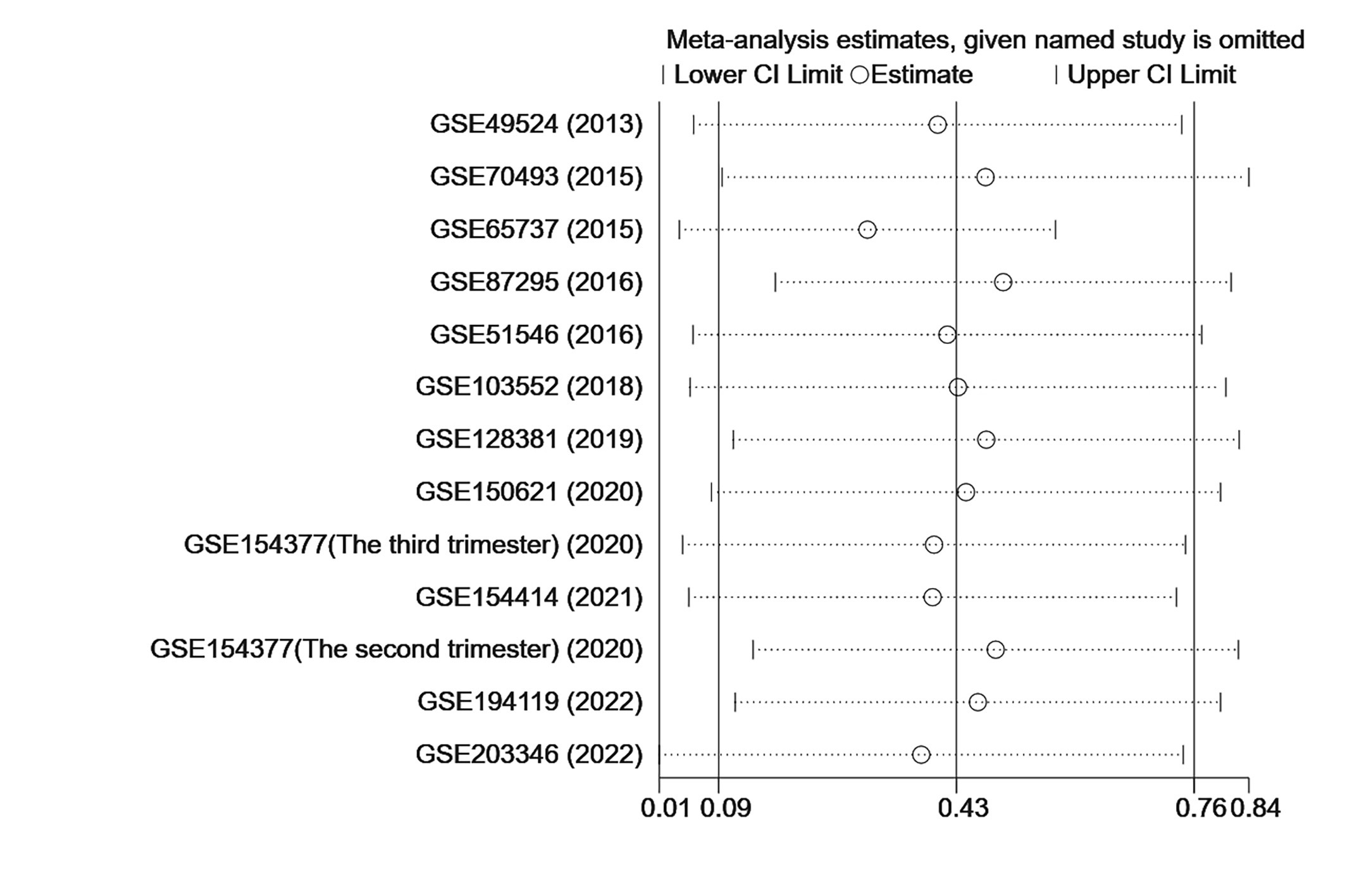

Supplement: Supplementary Figure 1 — Begg’s funnel plot for the assessment of potential publication bias in the samples (A: mRNA; B: protein). [file Image_1.jpeg]

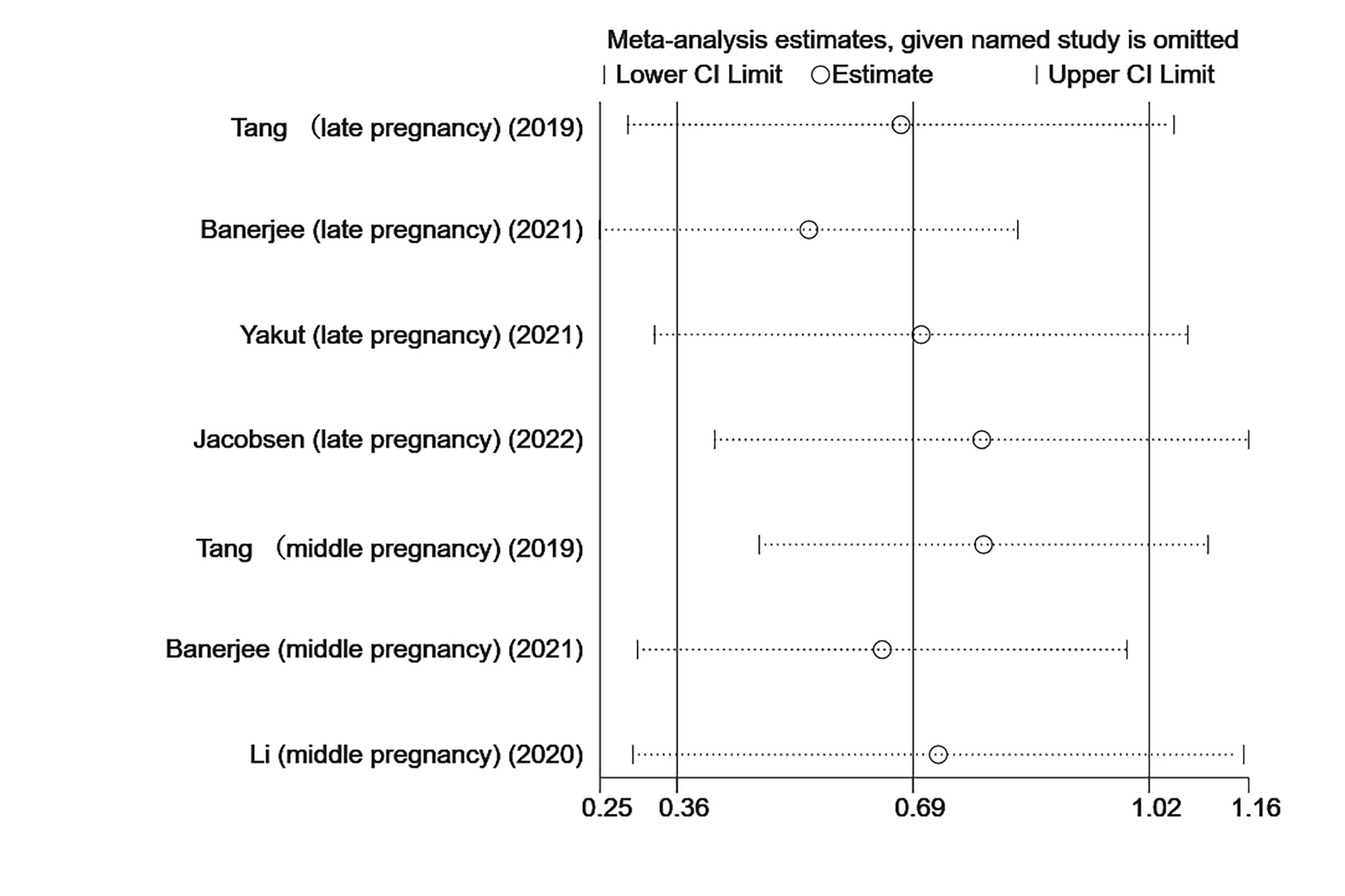

Supplement: Supplementary Figure 2 — Sensitivity test of GDF-15 expression between GDM patients and non-GDM pregnant women (A: mRNA; B: protein). [file Image_2.jpeg]

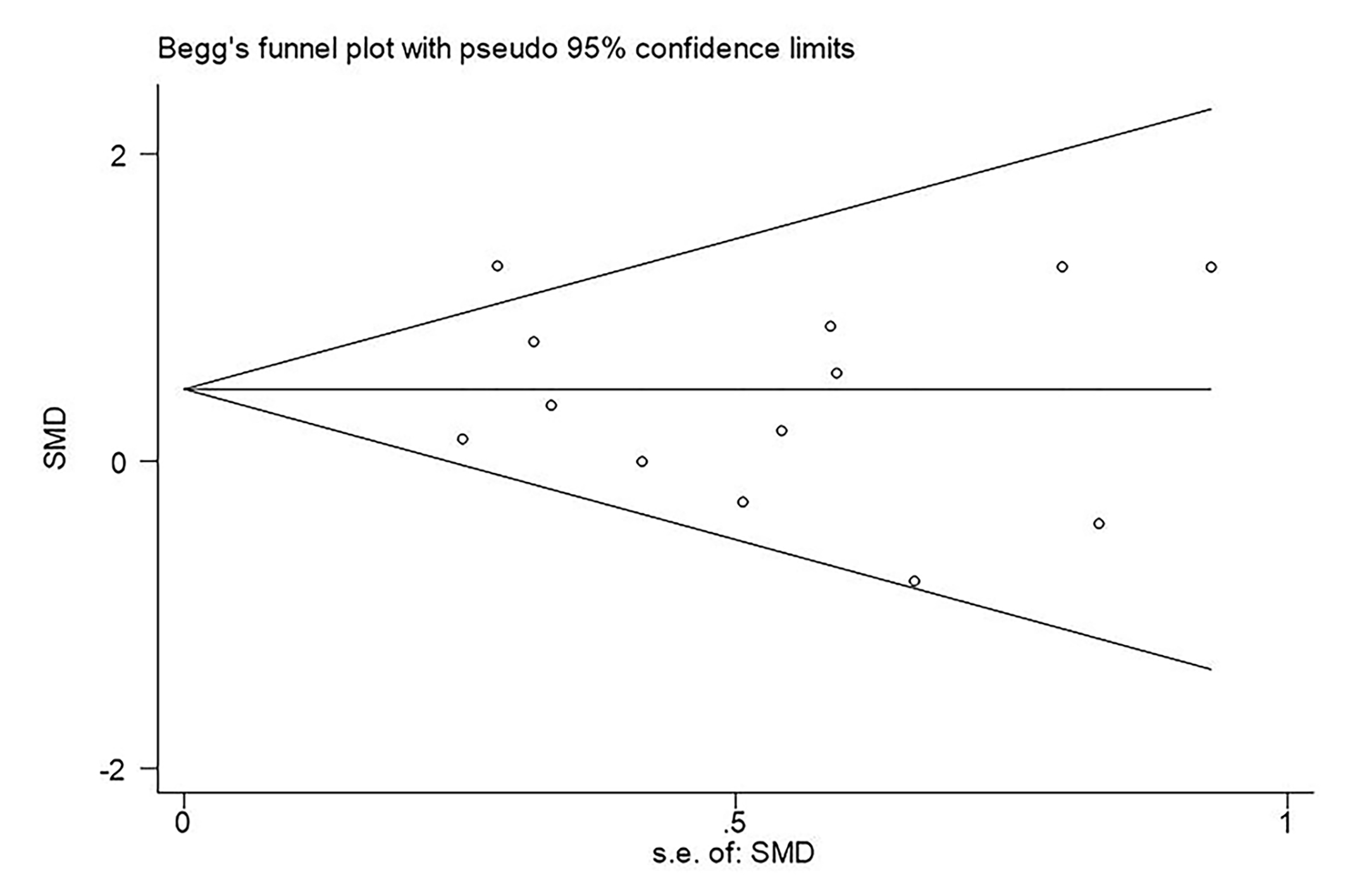

Supplement: Supplementary Figure 3 — Difference of GDF-15 mRNA expression between GDM patients and non-GDM pregnant women (excluding preeclampsia or other diseases that may influence GDF-15 expression). [file Image_3.jpeg]

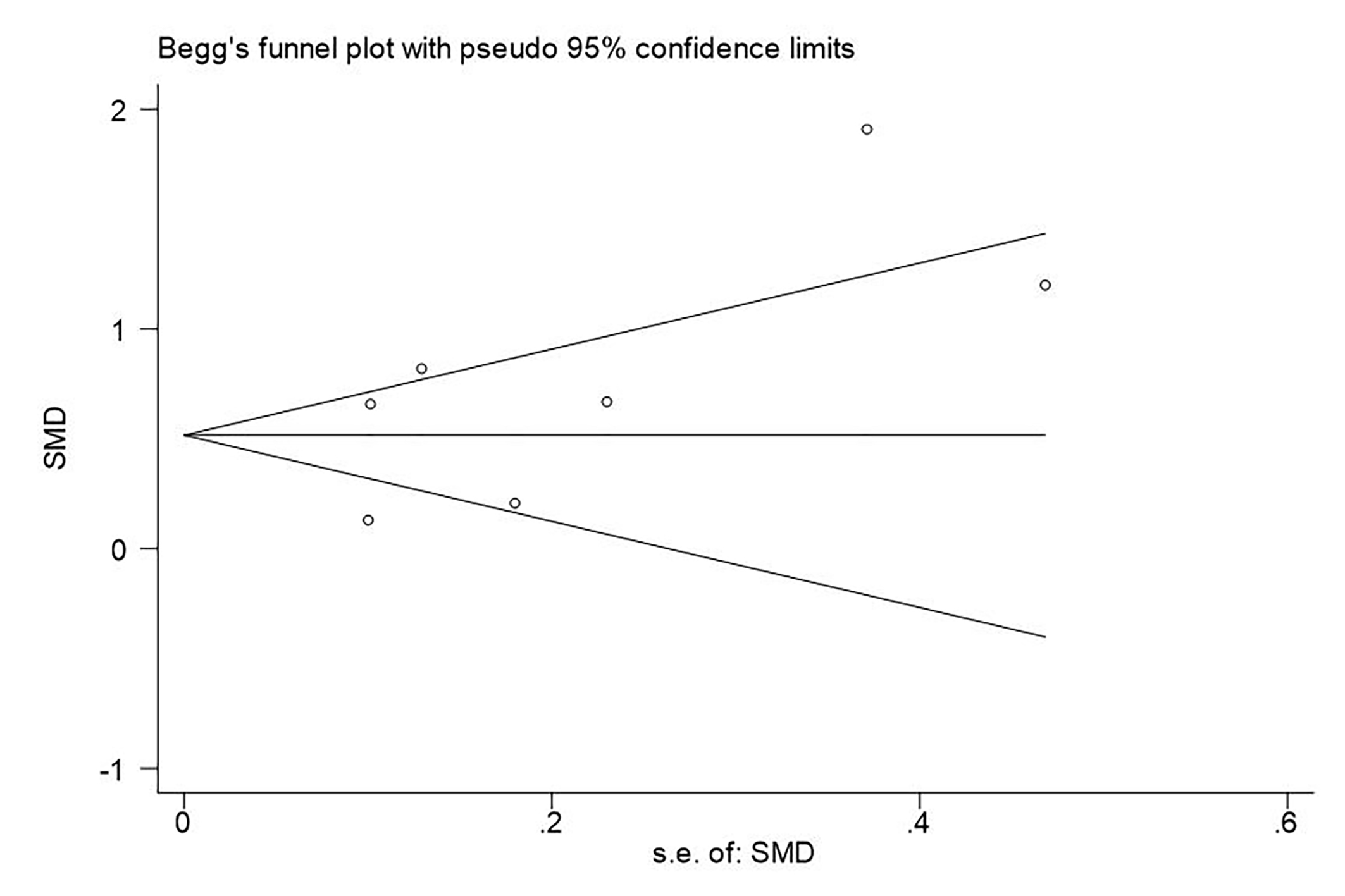

Supplement: Supplementary Figure 4 — Difference of GDF-15 protein expression between GDM patients and non-GDM pregnant women (excluding preeclampsia or other diseases that may influence GDF-15 expression). [file Image_4.jpeg]
