# Supplementary material for: Association between growth differentiation factor 15 levels and gestational diabetes mellitus: A combined analysis
Source: Front Endocrinol (Lausanne). 2023 Jan 20;14:1084896. doi: 10.3389/fendo.2023.1084896 (PMC9895392; doi:10.3389/fendo.2023.1084896)
Supplement: Supplementary file 5 [file Table_1.docx]

Supplementary Table 1. Exclusion criteria for studies.

| Dataset | Exclusion criteria for case | Exclusion criteria for control | Excluding & |
| --- | --- | --- | --- |
| GSE49524 | 3 Caucasian GDM women (GDM was diagnosed not later than 28th gestational week and GDM women fully recovered after delivery) | Women randomly selected matching for age (35.6 ± 4.0 and 34.4 ± 4.5, respectively) and pre-gestational weight (kg, 62.1 ± 13.7 and 67.9 ± 29.3). Basic anthropometric parameters did not differ significantly between the groups: height (mt)1.66 ± 0.04 (C) and 1.62 ± 0.09 (GD) and pre-gestational body mass index: 22.5 ± 3.7 (C) and 25.5 ± 2.4 (GD). | Not mentioned |
| GSE87295 | NA | NA | NA |
| GSE51546 | NA | NA | NA |
| GSE150621 | NA | NA | NA |
| GSE154414 | NA | NA | NA |
| GSE194119 | NA | NA | NA |
| GSE70493 | Placenta samples were selected among mothers with a clinical diagnosis of gestational diabetes, excluding women with preexisting hypertension or pre-existing diabetes mellitus. | “Controls” were identified among samples with no gestational diabetes, no pregnancy-induced hypertension, no preeclampsia, and no previous hypertension or diabetes. | Yes |
| GSE65737 | The exclusion criteria for mothers were as follows: multiple pregnancies, hypertensive disorders, history of diabetes prior to conception, polycystic ovary syndrome, Cushing’s syndrome, pheochromocytoma, acromegaly, history of smoking, chemical dependency, use of assisted reproductive technology, multiple gestation, and any other confounding pathologies, including hyperthyroidism and hypothyroidism. The exclusion criteria for infants were as follows: preterm birth, fetal growth restriction, fetal congenital anomalies, and other confounding pathologies, such as neonatal infection. | | Yes |
| GSE128381 | The dataset clearly indicated whether each subject had gestational diabetes mellitus or not, and whether each subject had preeclampsia or not. | | Yes |
| GSE154377 | Of the 160 subjects who have delivered, there were 99 normal pregnancies and 61 with APOs. These APOs were divided in IPD (ischaemic placental disease) (n = 37), consisting of preeclampsia (PreX), gestational hypertension (gHTN) and intrauterine growth restriction (IUGR), IPD with gestational diabetes (GDM) n = 4; IPD with chronic hypertension (cHTN) n = 3; cHTN only n = 11 and GDM only n = 13. We excluded the subjects with cHTN from the early DNA and RNA biomarker detection, although included in the pre-pregnancy body mass index (BMI) analyses. | | Yes |
| GSE203346 | We excluded the pregnant women with the following conditions from our study: (1) having chronic diabetes complicated with pregnancy or preeclampsia. (2) suffering from liver or kidney dysfunctions, or other chronic diseases which required long-term drug treatment; (3) showing a mental disorder or a serious infection; (4) smoking, drinking and drug abuse during pregnancy; (5) fetal chromosome abnormalities; (6) lacking registration information in the Women’s and Children’s Insurance Systems. | | Yes |

NA, not available

&, preeclampsia or other diseases that may influence GDF-15 expression

| Author | Article | Exclusion criteria | Excluding & |
| --- | --- | --- | --- |
| Tang | Serum growth differentiation factor 15 is associated with glucose metabolism in the third trimester in Chinese pregnant women | Women with autoimmune disease, thyroid disease, heart trouble, liver or kidney disease, tumors, hematopathy, and other known diseases affecting glucolipid metabolism or serum levels of GDF15 were excluded from this study. | Yes |
| Banerjee | A study of serum growth differentiation factor 15 in Indian women with and without gestational diabetes mellitus in the third trimester of pregnancy and its association with pro-inflammatory markers and glucose metabolism | None of the participants were smokers, and the subjects with inflammatory bowel conditions, major systemic illness, hypothyroidism, and twin pregnancy were excluded. | NA |
| Yakut | Is GDF-15 level associated with gestational diabetes mellitus and adverse perinatal outcomes? | Exclusion criteria for the participants included having a history of chronic systemic disease, pregestational diabetes mellitus, fetal congenital abnormalities, corticosteroid administration, tocolysis and multiple gestations. GDM with no medication (only diet and lifestyle modifications) and pregnancy complications such as chorioamnionitis, fetal growth restriction, premature preterm rupture of pregnancy and preeclampsia. | Yes |
| Jacobsen | Cardiovascular biomarkers in pregnancy with diabetes and associations to glucose control | Only women with singleton pregnancies, and no history of hypertension or other inflammatory diseases (e.g., autoimmunity or cancer) were included. | Yes |
| Li | Serum growth differentiation factor 15 is closely associated with metabolic abnormalities in Chinese pregnant women | This study excluded participants with autoimmune disease, liver or kidney disease, blood system disease, thyroid disease, heart disease, tumor and other diseases known to affect glucose and lipid metabolism or serum GDF15 concentrations. | Yes |

NA, not available

&, preeclampsia or other diseases that may influence GDF-15 expression
